# Arterial Emboli Complicating Cisplatin Therapy

**DOI:** 10.1155/2012/276385

**Published:** 2012-02-28

**Authors:** Campbell D. Tait, Elaine M. Rankin

**Affiliations:** Division of Cancer Medicine, Ninewells Hospital and Medical School, University of Dundee, Dundee DD1 9SY, UK

## Abstract

We report three cases of arterial emboli in patients with lung cancer treated with cisplatin chemotherapy. All three patients were managed without surgical intervention but subsequent oncological treatment was complicated by the sequelae of arterial emboli. We discuss the issues surrounding these patients and the importance of identifying patients at risk of arterial embolic phenomena with cisplatin treatment.

## 1. Introduction

Cisplatin is a cytotoxic drug used in the treatment of several tumour types including lung, bladder, testicular, and ovarian cancer, with favourable outcomes and tolerable side effects in many cases. Side effects noted by the British National Formulary include nephrotoxicity, ototoxicity, peripheral neuropathy, hypomagnesaemia, and myelosuppression, in addition to nausea and vomiting [1]. Arterial embolism is not a recognised side effect as such, although correlations have been made with vascular events and cisplatin-based chemotherapy.

Factors that may be associated with these vascular events include hypomagnesaemia-induced arteriolar constriction and raised levels of von Willebrand factor [2–4]. Patients with lung cancer have a higher incidence of thromboembolic events [5], although these are usually due to clots in the venous system. Chemotherapy further increases this risk [5]. Arterial emboli have been reported as complications of treatment, which has included cisplatin in a few isolated case reports [6–9]. We describe three patients with lung cancer in whom arterial emboli were precipitated by cisplatin chemotherapy and address the issues of management in these challenging situations.

## 2. Case Presentation

### 2.1. Patient 1

This 60-year-old woman with T_x_N_3_M_0_ small cell lung cancer was treated with cisplatin/etoposide. She had a past medical history of varicose veins and was a smoker of 45 pack years. One week after her second cycle of treatment, she complained of pain and numbness in her right hand, which became cold. CT angiography revealed multiple aortic thrombi, a nonocclusive thrombus at the bifurcation of the brachiocephalic artery and proximal subclavian artery stenosis, with partial retrograde flow in the vertebral artery. Radial and ulnar arteries were occluded several centimetres to the wrist. In light of these findings, the patient was treated with treatment-dose low-molecular-weight heparin (LMWH) on an outpatient basis. In addition, cisplatin was switched to carboplatin for subsequent cycles, with LMWH cover. Subsequently, the circulation in her hand improved, although she now has a slightly diminished right radial pulse.

### 2.2. Patient 2

This 60-year-old woman, who was a 25 pack year ex smoker, was treated with cisplatin and docetaxel for T_4_N_3_M_0_ adenocarcinoma of the lung. 6 days after her second cycle of treatment, she developed acute onset lower abdominal pain with associated excruciating pain in both feet, right worse than left. On examination, her feet were light blue in colour, with absent dorsalis pedis and posterior tibial pulses bilaterally. A nonocclusive thrombus in the thoracic aorta was apparent on subsequent CT angiography, with occlusion of anterior and posterior tibial arteries bilaterally. Her chemotherapy regime was changed to gemcitabine in combination with carboplatin, and she was given treatment dose of LMWH. The patient responded well to conservative management and regained adequate perfusion in both feet.

### 2.3. Patient 3

This 53-year-old woman, who was a 60 pack year smoker with longstanding bilateral thigh and buttock claudication at 20–30 yards on flat ground, was treated with one dose of cisplatin and pemetrexed for T_4_N_1_M_0_ adenocarcinoma. She was admitted 11 days later in extremis with ischaemic legs and severe sepsis and found to have distal aortic occlusion, on a background of chronic aorto-iliac claudication. At presentation, no pulses distal to her abdominal aorta were palpable. Treatment in this case again consisted of LMWH and transfer to high dependency for inotropic support. Had the anaesthetic risk not been so high, she would have undergone aortobifemoral bypass graft. Despite conservative measures, this patient arrested and died.

## 3. Discussion

Several key issues have arisen after the treatment of the above three patients, as we are now more aware of the potential sequelae of cisplatin therapy for treatment of lung cancer.

Arterial emboli in these circumstances can present with subtle or florid symptoms, as we have found with the above patients. One should have a high index of suspicion of arterial embolic disease, when patients on cisplatin develop any new onset vascular symptoms or signs which lie on the spectrum of pallor, paraesthesia, pain, pulselessness, paralysis, and that of becoming perishingly cold.

It has to be considered whether lung cancer patients in particular are thus more susceptible to the development of arterial emboli when treated with cisplatin. As all three of our patients were managed conservatively with treatment dose dalteparin, it is useful to be aware that surgical intervention is often unnecessary. As ever, the patient in context must be considered and sweeping generalisations regarding diminished life expectancy should not negate intervention. Quality of life and the preservation of adequate limb perfusion are paramount.

Patients treated with cisplatin who develop vascular symptoms, particularly on a background of atherosclerosis, warrant urgent examination of the affected limb, with comparison made with the contralateral limb. Proper documentation of findings is vital, so response to treatment can be gauged accurately. Vascular history should be taken into account, although it may be the case that the patient has no risk factors other than a diagnosis of cancer [9]. It is reasonable to deduce that vascular risk should be identified prior to starting treatment with cisplatin therapy, in order to assess which patients may be at greater threat of arterial embolic complications. Having a lower threshold for assessment with angiography would perhaps be useful in the early investigation of these patients.

The issue of prophylactic anticoagulant therapy for all lung cancer patients treated with cisplatin chemotherapy needs to be addressed. This would reduce the risk of thromboembolic disease in any case, particularly in less mobile, arteriopathic, and procoagulable patients who are already at an increased risk due to underlying malignancy in any case [5]. If one thrombotic event occurs with cisplatin, should this preclude further treatment with the same chemotherapeutic regime? Arterial embolus is an important complication of cisplatin therapy to be aware of, but one which can often be managed successfully with conservative treatment on the ward, and one which will hopefully cause minimal interruption to the patient if recognised and dealt with promptly.
